# Method Comparison for Enhanced Recovery, Isolation and Qualitative Detection of *C. jejuni* and *C. coli* from Wastewater Effluent Samples

**DOI:** 10.3390/ijerph120302749

**Published:** 2015-03-02

**Authors:** María Ugarte-Ruiz, Diego Florez-Cuadrado, Trudy M. Wassenaar, María Concepción Porrero, Lucas Domínguez

**Affiliations:** 1 VISAVET Health Surveillance Centre, Complutense University of Madrid, Avda. Puerta de Hierro, s/n. 28040 Madrid, Spain; E-Mails: maria.ugarte@visavet.ucm.es (M.U.-R.); d.florez@visavet.es (D.F.-C.); cporrero@visavet.ucm.es (M.C.P.); lucasdo@visavet.ucm.es (L.D.); 2 Animal Health Department, Veterinary Faculty, Complutense University of Madrid, Avda. Puerta de Hierro, s/n. 28040 Madrid, Spain; 3 Molecular Microbiology and Genomics Consultants, Zotzenheim 55576, Germany

**Keywords:** *Campylobacter*, urban effluents, isolation protocols, antimicrobial resistance

## Abstract

Seeking a sensitive protocol, culture-dependent methods were compared to detect thermophilic *Campylobacter* species in untreated urban effluents. We evaluated various combinations of selective media, with and without an enrichment steps, as well as an extra filtration step. Culture-independent real-time quantitative PCR was also included and all detected isolates underwent antimicrobial susceptibility testing. All tested water samples contained *Campylobacter* DNA, but only 64% were positive after culture. Although enrichment using Preston broth resulted in better recovery of potentially stressed *Campylobacter* than Bolton or Campyfood broth (CFB), there was no significant increase in efficiency compared to direct plating. The type of selective agar media used, on the other hand, had a significant effect, with CASA plates performing better than mCCDA or CFA ones. Inclusion of an enrichment step increased the ratio of *C. coli vs.* *C. jejuni* being isolated. Resistances against all antimicrobials tested were observed in *C. coli*, but fewer instances of resistance were found in *C. jejuni* isolates. Whether this difference was the result of selection during the enrichment step could not be determined. The presence of *Campylobacter* in urban effluents can be considered as a valuable proxy for *Campylobacter* populations present in urban environments.

## 1. Introduction

*Campylobacter* is one of the most common causes of food-borne bacterial disease in humans worldwide. In the European Union, the most commonly species detected associated with campylobacteriosis are *C. jejuni*, followed by *C. coli* [1,2]. These thermophilic species asymptomatically colonize the intestinal mucosa of a wide variety of birds and mammals, including food-producing animals [3,4], which explains their frequent encounter as zoonotic, food-borne pathogens. The observed increase in the proportion of *Campylobacter* isolates with reduced susceptibility to antimicrobials, particularly against quinolones and macrolides, is a cause of concern [5,6] as this could have serious potential consequences for public health [7,8].

Despite the fact that poultry products are considered the major source of *Campylobacter* infection, multiple other sources exist, including untreated drinking water [1,5]. *C. jejuni* and *C. coli* (collectively described here as *Campylobacter*) are often present in aquatic environments, which may provide a reservoir, or even a direct source, of *Campylobacter* infection [9]. Multiple outbreaks associated with drinking water have been described [10,11,12]. Recreation in surface waters or ingestion of raw fruits or vegetables that have been in contact with contaminated water pose recognized risks [13,14]. Waterborne *Campylobacter* spp. is assumed to originate from animal faeces, agricultural leaks or wastewater contamination. The organism can indeed be recovered from untreated wastewater [9,15,16,17,18]. However, the detection methods are rather insensitive and elaborate.

Efforts are ongoing to improve the methodology for detection and isolation of *Campylobacter*, with attempts to reduce isolation or confirmation t ime, increase sensitivity, and standardize procedures; although, it is unlikely that any single method would be optimal for all kind of samples, irrespective of their origin [19,20,21]. For water samples, isolation of *Campylobacter* is rarely successful, as the methodology is not sufficiently sensitive [22,23]. It should be noted that the media typically used for isolation were originally developed to be applied to stool and other clinical samples, which contain vastly higher numbers of viable *Campylobacter* cells than are present in environmental samples [12,24]. 

An enrichment step is often necessary to recover low numbers or potentially damaged cells, as is typically the case with wastewater samples. However, background bacteria also proliferate during enrichment culture, and often grow at a rate faster than *Campylobacter*. *Campylobacter* enrichment media typically contain oxygen quenching agents to neutralize the adverse effects of toxic oxygen species, as well as selective agents such as cefoperazone, polymyxin B, rifampicin or trimethoprim to reduce background flora [19,25]. These latter supplements may actually reduce the isolation of damaged *Campylobacter* cells, since particular strains or *Campylobacter* species can be sensitive to these antibiotics [22,24].

To gain insights into the presence of *Campylobacter* in urban effluents, we initiated a study to compare the performance of different culture-dependent methods for its detection in urban effluent samples obtained from a wastewater treatment plant (WWTP). In view of the difficulty with detection of *Campylobacter* in water samples that typically contain a highly variable background flora, we decided to compare real samples, rather than perform a standardized comparison with artificially spiked samples. Effluent wastewater samples were also tested with real time quantitative PCR (qPCR) and the strains obtained were characterized by antimicrobial susceptibility testing. All the samples were found positive using qPCR, while culture alone recovered *Campylobacter* in 64% of the samples. Direct plating on CASA agar was a fast and reliable method for *Campylobacter* isolation and performed better than the recommended mCCDA plates. Addition of a filtration step or enrichment in Preston media could improve the chance of recovering *Campylobacter*, however, the method of choice influences the recovered species (*C. jejuni vs. C. coli*).

## 2. Material and Methods

### 2.1. Sampling Collection

Fifty samples of untreated urban effluents were taken from November 2010 to November 2013 from a wastewater treatment plant collecting water from a city in the center of Spain. All samples were collected in sterile containers and transported to the laboratory where culture was performed immediately after reception. In addition, an aliquot of each fresh sample was stored at 4 °C for subsequent DNA extraction and qPCR.

### 2.2. Culture-Dependent Detection by Direct Plating and Enrichment

Three selective plates were compared by direct culture: blood-free modified charcoal Cefoperazone Deoxycholate agar (mCCDA, PO5091A, Oxoid, Basingstoke, UK), chromogenic-like Campyfood agar (CFA, Ref 43471, bioMérieux, Marcy l´Etoile, France) and selective chromogenic medium CASA (AEB520270, AES Chemunex, Marcy l´Etoile, France). These selective plates were also combined with an enrichment step using one of three enrichment broths in all possible combinations (Table 1): Bolton, Preston (both from Oxoid) and Campyfood broth (CFB, bioMérieux). 

For direct plating, a swab was dipped into the homogeneous sample (25 mL) and streaked onto selective plates. Following incubation at 42 °C for 48 h under microaerobic atmosphere (Campygen, Oxoid), the plates were examined. Regarding enrichment, Bolton broth (CM0983, Oxoid) was added antibiotic supplement (SR0183, Oxoid) and 5% lysed horse blood (SR0048, Oxoid); while Preston broth (Nutrient broth N° 2, CM0067, Oxoid) was supplemented with 5% lysed horse blood (SR0048, Oxoid) and antibiotic (SR0204 and SR0232E, Oxoid). CFB broth (Ref 42643, bioMérieux) was bought commercially and ready-to-use. A volume of 25 ml of urban effluents samples were transferred to sterile stomacher bags with filter and pouch and mixed with 225 mL of each enrichment broth. These were incubated with a Genbox atmosphere generator (bioMérieux). Enrichment was performed for 4–6 h at 37 °C followed by 48 h at 42 °C for Bolton broth and 48 h at 42 °C for Preston broth and CFB. After this incubation step, 200 µL were cultured for 48 h on the selective agar plates (mCCDA, CFA, CASA) as described above. 

In this analysis, the volume of urban effluents tested was 25 mL for each protocol. In addition, 27 water samples were pre-filtrated before culturing onto selective agar or enrichment broths as recommended in ISO 17995:2005 [26]. For this purpose, 25 mL of water was passed through filters of 0.22 μm (Nalgene, Thermo Fisher Scientific, Waltham, MA, USA), after which the membrane was either directly added to the enrichment broth or streaked out onto a selective plate. 

All colonies with a *Campylobacter*-typical morphology (according to the manufacturer's instruction for each plate type) were cultured onto blood agar plates (bioMérieux) at 37 °C for 48 h in microaerobic atmosphere (Campygen, Oxoid). For further identification, conventional PCR to amplify a gene specific for *C. jejuni*, one for *C. coli*, and a genus-specific 16S rRNA fragment was used, as previously described [27]. When more than one colony morphology was observed, representative colonies of different morphologies were picked. A sample was considered positive if at least one colony was confirmed by PCR as *C. coli*, *C. jejuni* or *Campylobacter* spp.

### 2.3. Culture-Independent Detection by Quantitative Real-Time PCR (qPCR)

**Figure 1:**
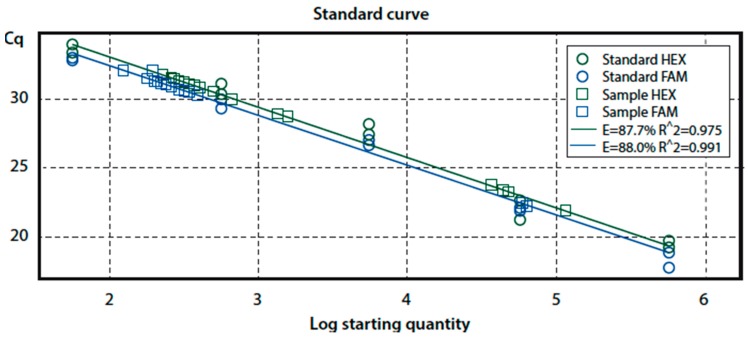
Linear regression curve of qPCR including the standards along with unknown sewage samples.

Culture-independent qPCR was carried out to confirm presence or absence of *Campylobacter* DNA in each water sample. Before DNA extraction, five aliquots containing 1.5 mL each of urban effluent were concentrated by centrifugation (15,500 g for 12 min). After removal of the supernatant, a further 1.5 mL of the original sample was added and centrifugation was repeated. The five precipitates thus obtained were resuspended each in 80 μL of the original supernatant and then combined. DNA was extracted from this concentrated suspension using a QIAamp DNA stool mini kit 50 (Qiagen, Hilden, Germany) and subjected to an in-house multiplex qPCR assay as previously described [27]. Reactions (final volume 25 µL) contained 5 µL of template DNA, 12.5 µL of QuantiTect Multiplex PCR No ROX Mastermix (Qiagen), 0.4 µM of each amplification primer, and 0.25 µM of each probe. The thermal cycle protocol included initial denaturation at 95 °C for 15 min, followed by 40 cycles (94 °C for 1 min, 56 °C for 1 min) and a final extension at 72 °C for 10 min. In order to generate a standard curve **(**Figure 1), 1 ng DNA of *C. jejuni* ATCC 33560 was mixed with 1 ng DNA of *C. coli* CRL C 2.2, and ten-fold serial dilutions were produced up to 10^−4^ (range: 5.649 × 10^5^ to 5.649 × 10^1^ DNA copies). All standard dilutions and samples were performed in triplicate. Fluorophore-linked probes and primers sequences used were:

*C. jejuni*
  HEX-5ʹ-AGATCCTATTTATGCTGCTTCTTTRC-BHQ
  JEJ1 (5ʹ -GGTGGTCATGGAAGTGCT)
  JEJ2 (5ʹ-CTCCTATGCTTACAACTGCTG)
*C. coli*
  FAM-5ʹ-ATAAAGTTGCAGGAGTTCCAGCTAAA-BHQ
  COL1 (5ʹ-ACTTTCCATGCCCTAAGAC)
  COL2 (5ʹ-TCCACCTATACTAGGCTTGTC)

### 2.4. Identification of Isolates Using 16S rRNA and MALDI-TOF

Isolates that were negative for the species-specific *C. jejuni* and *C. coli* PCR but positive for the *Campylobacter* genus PCR (*n* = 4) were further characterized by 16S rRNA PCR. Amplicons produced with universal 16S rRNA primers (described in Baliarda *et al.* [28]), were purified using the QIAquick PCR Purification kit (Qiagen) and externally sequenced (Stabvida, Lisbon, Portugal). All 16S rRNA sequences were compared to GenBank entries [29] and homologies to most closely related sequences were determined using ClustalW [30]. In addition, calculations of pair-wise 16S rRNA gene sequence similarities were achieved using the EzTaxon server [31].

A selection of 18 isolates was further characterized using MALDI-TOF/TOF mass spectrom*etry*. The bacteria were directly spotted onto a polished steel target plate using a saturated solution of α-HCCA (Bruker Daltonics, Bremen, Germany) dissolved in 50% acetonitrile, 47.5% water and 2.5% trifluoroacetic acid (Fluka, Sigma-Aldrich, St. Louis, MO, USA). Mass spectra acquisition and analysis was performed on a Bruker UltraFlextrem platform (Bruker Daltonics) using MALDI Biotyper™ 3.0 software (Bruker Daltonics) using default settings.

### 2.5. Antimicrobial Susceptibility Testing

Antimicrobial susceptibility testing was performed using broth microdilution towards ciprofloxacin, erythromycin, gentamicin, tetracycline, streptomycin and nalidixic acid (Table 2). Isolates were grown on blood agar plates (bioMérieux) and incubated for 48 h at 37 °C at microaerophilic conditions (Oxoid). Collected cultures were added to 11 mL of cation-adjusted Mueller-Hinton broth with TES (TREK Diagnostics Systems, Waltham, MA, USA), standardized to 0.5 McFarland turbidity, and supplemented with 600 μL of lysed horse blood freshly prepared in house from defibrinated horse blood (Oxoid). This mix was distributed onto EUCAMP2 microdilution plates (TREK Diagnostics Systems) and incubated under microaerophilic conditions for 48 h at 37 °C. *C. jejuni* strain ATCC 33560 was used as a quality control. The interpretation of the quantitative data was performed as described by the European Committee of Antimicrobial Susceptibility Testing, EUCAST [32].

### 2.6. Data Analysis

Data were analyzed using SPSS (19.0 IBM, Armonk, NY, USA). Statistical significance of differences (*p*-value < 0.05) was assessed by Fisher’s exact test using the WinPepi software (11.25 version, Abramson [33]). For multiple comparisons, Holm correction was applied with R Software (R Development Core Team 3.0.2, 2013). Confidence Intervals (CI) at 95% were calculated for a subset of 24 samples that were tested by all methods, using the online tool developed by WinEpi [34].

For the analysis of MALDI-TOF results, the scores obtained were contrast-based as per the manufacturer's recommendations. An identification log (score) value ranging from 0 to 3 indicated the pattern-matching extent according to the specifications of the Biotyper system. Score values of 0 to 1.699 generally indicated no reliable identification; score values of 1.7 to 1.999 indicated probable genus identification; score values of 2.0 to 2.299 indicated secure genus identification and probable species identification; and score values of 2.300 to 3.000 indicated highly probable species identification.

## 3. Results

### 3.1. Comparison of Campylobacter Detection by All Tested Methods

Irrespective of which protocol was used for isolation, 32 of 50 samples (64%) were found positive by culture-dependent methods, while qPCR detected *Campylobacter* DNA in all of the samples. The results of the culture-dependent detection methods, regardless of the *Campylobacter* species detected, are summarized in Table 1.

**Table 1:** Detection of *Campylobacter* spp. by culture-dependent methods.

Method		Nr. of Positive Samples/nº Samples Tested (all Plates)	Per Selective Agar Plate		
			mCCDA	CFA	CASA ^a^
Direct plating		14/50	0/50	5/50	10/24
Enrichment (all broths)		19/50	10/50	12/50	10/24
Per enrichment broth	Bolton	2/50	0/50	0/50	2/24
	Preston	18/50	10/50	12/50	9/24
	CFB	0/50	0/50	0/50	0/24
Filtration ^c^, direct plating		9/27	0/27	0/27	9/24
Filtration ^c^, enrichment		8/27	0/27	3 ^b^/27	6 ^b^/24

^a^ Only 24 samples were incubated on CASA plates; ^b^ Enrichment broth was Preston in all positive cases; ^c^ Filtration was added as an extra step in 27 samples.

For the investigated samples, direct plating on CASA was the best-performing isolation method, followed by Preston enrichment combined with CASA agar plates. Although the difference between these two methods was not statistically significant, direct plating was faster, simpler and cheaper than the method involving enrichment. When resources are limited or large numbers of samples need to be analysed, direct plating on CASA plates would therefore be preferable. For optimal coverage, however, filtering and Preston enrichment could be added as extra steps prior to CASA plating, separately and in combination.

### 3.2. Congruity of All Culture-Dependent Methods

Table 1 includes nine protocols that did not yield any positive samples. Excluding these, there remained 12 protocols that resulted in positive findings, and the congruity of these was assessed for the 24 samples that had been checked using all these methods. Of these, 21 samples were positive by one or more method, whereby culture on CASA plates (with and without combined Preston enrichment) recovered *Campylobacter* in 17 cases (81%; CI 95%: 64–98). This is the highest number of positives obtained by any combination of two methods (for comparison: CASA direct plating only identified 10 and Preston with CASA produced nine positive samples). One of the 21 water samples was positive by six methods and two by five. At the other extreme, 10 samples were positive by one protocol only, and in every one of these 10 cases, the final culture had been performed on CASA plates, though in seven cases in combination with enrichment, filtering, or both. When combining all protocols with CASA plates as the final step, not one sample was reported negative that was positively cultured on an alternative plate media, which again identifies CASA plates as superior to CFA or mCCDA. For optimal coverage, filtering could be added, which added two exclusively positive sample using direct plating, Preston enrichment (adding three exclusively positive sample), and Preston combined with filtering, adding one exclusively positive when combined with CASA plating.

### 3.3. Campylobacter Species Isolated from Effluent Water

All obtained *Campylobacter* isolates were identified individually using multiplex PCR. A total of 77 colonies were thus speciated, resulting in 53 *C. coli*, 20 *C. jejuni* and four non-*jejuni*, non-*coli* that were subsequently identified as *Arcobacter butzleri* (see below). For 18 samples, more than one colony was analysed separately. Twelve of these samples were found to contain a mixture of either *C. jejuni*, *C. coli* or *Arcobacter butzleri*. A higher proportion of samples was positive for *C. coli* than for *C. jejuni* and this difference was significant (*p* < 0.05). Considering only the subset of 24 samples tested using all protocols, from 16 of these *C. coli* was cultivated. Direct plating on CASA produced *C. coli* in two samples (12%; CI 95%: 0%–29%), adding filtration increased this to 6 (37%; CI 95%: 14%–61%) and the combination of Preston enrichment with CASA resulted in 8 *C. coli* positive samples (50%; CI 95%: 25%–74%). The other *C. coli* isolates were obtained with the alternative enrichment broths. *C. jejuni*, on the other hand, was more often detected by direct plating, in particular on CASA agar (with and without filtering), which obtained 11/12 (92%; CI 95%: 76%–100%) of the detected *C. jejuni* positive samples. The only other *C. jejuni* positive sample was derived from a CASA plate after Preston enrichment. If optimal recovery of both species were attempted with minimal experimental investment, direct plating on CASA with and without Preston enrichment would detect 10/16 *C. coli* (62%; CI 95%: 39%–86%) and 9/12 *C. jejuni* (75%; CI 95%: 50%–99%).

The culture-independent qPCR found one sample positive for *C. coli* exclusively, while DNA of both species was detected in all other samples. Most samples contained *Campylobacter* DNA in the range of 10^3^ to 10^4^ copies/mL, and there was no correlation between this detected DNA concentration and the number of culture-dependent methods reporting presence of viable cells.

Four isolates were obtained from four different water samples that were negative for the PCR probes specific for *C. jejuni* and *C. coli* genes nevertheless produced a genus-specific 16S amplicon. Their complete 16S rRNA gene was amplified and sequenced for further identification. Comparative sequence analysis to sequences stored in GenBank revealed the highest similarity to the type strain *Arcobacter butzleri* NCTC 49616 and *A. butzleri* strain RM4018 [35,36] with homologies ranging between 98.7%–100%. The novel sequences were submitted to the European Nucleotide Archive with accession numbers LN811434, LN811435, LN811436, and LN811437 (*Arcobacter butzleri* partial 16S rRNA gene, from isolates ZTA11/01227CPF, ZTA11/00338CPF, ZTA11/00429CPF, and ZTA13/02375CASA_FILTRO, respectively).

### 3.4. Identification of Isolates Using MALDI-TOF

The four presumed *A. butzleri* isolates were further characterized by MALDI-TOF, and for comparison 14 *Campylobacter* (six *C. jejuni* and eight *C. coli*) isolates were also included. The MALDI-TOF results confirmed the speciation by PCR in all *C. coli* and *C. jejuni* cases and 16S rRNA PCR in all *Arcobacter butzleri* isolates. Score values in the range of 2.083–2.281 (three isolates) and 2.363 (one isolate) were interpreted as *A. butzleri* species identification. There was no correlation between sequence similarity to the *A. butzleri* type strain NCTC 49616 and MALDI-TOF scores; for instance, the isolate whose amplicon produced the lowest similarity (98.71%) produced a score value of 2.259. The score values of *C. coli* and *C. jejuni* isolates ranged between 2.303–2.428 in most cases, but for four isolates that produced score values between 2.039 and 2.218.

### 3.5. Results of Antimicrobial Susceptibility Testing

Sixty two of the 77 isolates obtained (51 C*. coli* and 11 *C. jejuni*) were analysed using susceptibility tests against six antimicrobials. The highest proportion of antimicrobial resistance was observed towards ciprofloxacin, to which 90% (CI 95%: 82%–98%) of all tested isolates found resistant, with no difference between *C. jejuni* and *C. coli.* Similar resistances were found for nalidixic acid, which is not surprising, as both might be the result of a common resistance mechanism. Lower percentages were found for tetracycline (84%; CI 95%: 75%–93% of total), with significant differences between the species (94%; CI 95%: 88%–100% of *C. coli* and 36%; CI 95%: 8%–65% of *C. jejuni*). Lower incidence still was found for gentamicin and erythromycin resistance in *C. coli*, (10%; CI 95%: 2%–18% and 16%; CI 95%: 6%–26% of the tested isolates, respectively), while all *C. jejuni* isolates were susceptible to these two antimicrobials. The higher incidence of resistance in *C. coli* compared to *C. jejuni* was statistically significant for streptomycin and tetracycline only (*p* < 0.001). In the majority of the cases (7/8; 87%, CI 95%: 65%–100%) when a sample produced more than one isolate, the antimicrobial resistance pattern found for these isolates was different. The distribution found in MIC are shown in Table 2.

## 4. Discussion

The detection of *Campylobacter* in urban effluent water can be used as a global parameter to estimate the presence of *Campylobacter* in an urban environment, provided detection methods are sufficiently sensitive. When we compared various culture-dependent detection protocols, we found 64% of water samples being positive, whereas *Campylobacter* DNA was detectable in all water samples. Currently, the culture-dependent methods applied in water quality monitoring can be arduous and are rarely successful [12]. Conversely, molecular detection methods based on DNA amplification such as qPCR are faster and more sensitive for *Campylobacter* detection [37], however, the DNA detected may be from viable, non-cultivable or dead cells as well as from circulating DNA [12,37]. It is well documented that *Campylobacter* spp. may enter a viable but non-culturable state when exposed to adverse conditions, which can decrease the recoverability on laboratory media [38,39]. Indeed, despite qPCR positive results obtained, it was impossible to recover cultivable bacteria from 36% of the samples of our analysis. 

**Table 2:** Antimicrobial susceptibility of *C.coli* and *C.jejuni* isolates from urban effluents samples.

Antimicrobial Agents	Species	MIC Range (mg/L)	ECOFF (mg/L) ^a^	Number of Isolates with a MIC (mg/L) of:											Number of Resistant Strains (%)
				0.12	0.25	0.5	1	2	4	8	16	32	64	128	
Gentamicin	*C.coli*	0.12–16	2		11	34	1					5			5 (10)
	*C.jejuni*		2	6	4	1									0 (0)
Ciprofloxacin	*C.coli*	0.06–4	0.5	1	3	1				46					46 (90)
	*C.jejuni*		0.5	1					1	9					10 (91)
Tetracycline	*C.coli*	0.25–16	2				2	1				48			48 (94)
	*C.jejuni*		1		3	3	1			1		3			4 (36)
Erythromicin	*C.coli*	0.5–32	8			2	23	17	1			3	5		8 (16)
	*C.jejuni*		4			9	1	1							0 (0)
Nalidixic Acid	*C.coli*	2–64	16							4	1		2	44	46 (90)
	*C.jejuni*		16						1				4	6	10 (91)
Streptomycin	*C.coli*	1–16	4				6	10	2	1	1	31			33 (65)
	*C.jejuni*		4				10	1							0 (0)

^a^ EUCAST: *C.coli and C.jejuni* data from the EUCAST MIC distribution website last accessed 6 June 2014.

Enrichment can, at least in theory, enhance the growth of damaged and injured cells [22], while filtration may improve recovery of low numbers of cells. For water samples, the ISO 17995: 2005 standard recommends the use of membrane filtration [26], followed by parallel enrichment with Bolton and Preston broth. However, in our study, filtration, enrichment, or both, did not significantly improve the detection of *Campylobacter* in samples of urban effluents. That enrichment does not improve recovery from water samples was already reported by Rosef *et al.* [40]. However, in contrast to previously published observations that direct plating of water samples would be suboptimal [41]; we obtained satisfying results by direct plating on CASA plates. The highest numbers of positive samples with a combination of any two methods would be obtained by direct plating on CASA together with Preston enrichment followed by CASA agar. 

We deliberately performed this study with real wastewater samples, instead of a comparison of methods with spiked samples containing a known, artificially added number of viable *Campylobacter* cells. The latter approach would not have captured the difficulty with recovering injured organisms, or the complication that different water samples contain different types and quantities of background flora, as elaborately discussed by Jokinen *et al.* [24]. Injured *Campylobacter* cells also make a comparison of qPCR results and culture results difficult, since the first detects DNA from dead as well as live cells. The distinction between these two can be made by addition of propidium monoazide (PMA) [42] or ethidium monoazide (EMA) [43] to the PCR, which avoids amplification of DNA from dead cells. However, for *Campylobacter*, conflicting results have been described for such assays. Whereas Josefsen *et al.* reported such a distinction to be successful for chicken carcass rinses [37]; Pacholewicz *et al.* [44] described insufficient PMA effectivity for carcass samples containing more than 10^4^ dead cells. That work demonstrated conflicting results between PMA-qPCR and culture methods, suggesting that qPCR combined with PMA did not fully reduce the signal from dead cells in naturally contaminated samples. Likewise, in another study, EMA-qPCR failed to detect viable cells correctly in spiked water samples [45]. In view of these disappointing results, we did not perform qPCR in presence of these chelating agents. 

Nearly all samples (49/50 or 98%) contained *C. coli* and *C. jejuni* DNA in approximately the same quantity as determined by qPCR. Nevertheless, particular culture methods favoured the recovery of one species over another. *C.coli* was most often isolated after enrichment using Preston, followed by CASA plates, while direct plating on CASA agar recovered mostly *C. jejuni.* Various studies have shown that the method used for detection may influence both the yield and the genetic diversity of the population under study [9,17,46,47,48] but we point out that the ratio of species detected is also influenced by the method of detection. It has been reported that diversity in *Campylobacter* species may be affected by their survival characteristics under environmental conditions. Korhonen and Martikainen [49] observed that *C. jejuni* survived longer in cultivable form than *C. coli* in lake water. Moreover, Thomas *et al.* [50] described that *C. jejuni* is the species most frequently identified from surface waters, commonly associated with sewage water discharges. Likewise, Meinersmann *et al.* [51] as well as Khan *et al.* [52] detected a higher proportion of *C. jejuni* than *C. coli* from river water samples. Conversely, it was suggested that *C. coli* is possibly surviving better than *C. jejuni* under environmental circumstances [53]. Our results suggest that the use of different protocols improves the chance to detect both species from the same sample, thereby assessing the microbial diversity more accurately. Antimicrobial susceptibility data also support this hypothesis, as the majority of the strains derived from the same sample offered a different antimicrobial resistance profile. 

Despite of similar amounts of DNA being present for both *C. jejuni* and *C. coli* in each water sample analyzed, we were not always able to cultivate both species. These results would indicate that microbiological methods might have underestimated the real diversity of the sample. There is also a growing number of *Campylobacter* species, other than *C. coli* or *C. jejuni*, as well as related genera, being recognized as emerging human and animal pathogens. The prevalence of these, such as *A. butzleri*, is probably unknown because the detection methods used can favor the recovery of some species and these emergent bacteria are not identified to the species level [20,54].

It has been proposed that aquatic environments are involved in the dispersion and evolution of antimicrobial resistance in bacteria, and wastewater may play a particularly important role in these processes [55]. This was considered a possibility for methicillin resistant *Staphylococccus aureus* [56]. Denis *et al.* [57] observed that the majority of *C. coli* and *C. jejuni* obtained from contaminated river waters in France were susceptible to ciprofloxacin, erythromycin, tetracycline, gentamicin and streptomycin. However, our data show that the majority of *C. coli* and *C. jejuni* isolates were resistant towards quinolones and, to a lesser extent, to tetracycline. A smaller proportion of *C. coli* but none of the *C. jejuni* isolates exhibited resistance against streptomycin, erythromycin and gentamicin. Similar difference between these two species were recently reported for poultry isolates from Spain [58] and from human clinical isolates [59]. We therefore assume that our results can be taken as a proxy for the bacterial population found in an urban environment, although we cannot completely rule out that the enrichment step that favoured *C. coli* may have selected for resistance as well, as our data were inconclusive on this point. 

Finally, four strains considered as *Campylobacter* spp. by PCR were subsequently identified as *Arcobacter butzleri*. All these strains were isolated using direct plating protocols. *Arcobacter* spp. has been described as closely related and phenotypically similar to *Campylobacter*, and has been isolated from various water samples, including untreated sewage [60,61]. There is currently no standardized protocol for *Arcobacter* isolation; thus, conventional culturing methods for *Campylobacter* detection have been used to identify *Arcobacter* [20,62]. *Arcobacter* has been isolated from wastewater samples before [62] and this organism receives increasing attention as a potential cause of human illness [63]. It has been reported that the genus *Arcobacter* has become increasingly important in recent years because its members have been associated with human illness and fecal contamination by humans and animals [62]. At present, *A. butzleri, A. cryaerophilus* and *A. skirrowii* are considered the most common species of the genus as emergent enteropathogens and potential zoonotic agents [61,64].

## 5. Conclusions

In conclusion, the effectiveness of recovery of *Campylobacter* from effluent waters by culture depends on the method of isolation. Direct plating on CASA agar provides a quick, simple and reliable method and performed better than the recommended mCCDA plates. Addition of a filtration step or enrichment in Preston could improve the chance of recovering *Campylobacter*, however, the method of choice influences the recovered species (*C. jejuni vs. C. coli*).

## References

[1] EFSA (European Food Safety Authority), ECDC (European Centre for Disease Prevention and Control) (2014). The European Union summary report on trends and sources of zoonoses, zoonotic agents and food-borne outbreaks in 2012. EFSA J..

[2] Whiley H., van den Akker B., Giglio S., Bentham R. (2013). The role of environmental reservoirs in human campylobacteriosis. Int. J. Environ. Res. Public Health.

[3] Allos B.M. (2001). *Campylobacter jejuni* infections: Update on emerging issues and trends. Clin. Infect. Dis..

[4] Nichols G.L., Richardson J.F., Sheppard S.K., Lane C., Sarran C. (2012). *Campylobacter* epidemiology: A descriptive study reviewing 1 million cases in England and Wales between 1989 and 2011. BMJ Open.

[5] Epps S.V.R., Harvey R.B., Hume M.E., Phillips T.D., Anderson R.C., Nisbet D.J. (2013). Foodborne *Campylobacter*: Infections, metabolism, pathogenesis and reservoirs. Int. J. Environ. Res. Public Health.

[6] García-Migura L., Hendriksen R.S., Fraile L., Aarestrup F.M. (2014). Antimicrobial resistance of zoonotic and commensal bacteria in Europe: The missing link between consumption and resistance in veterinary medicine. Vet. Microbiol..

[7] Iovine N.M. (2013). Resistance mechanisms in *Campylobacter jejuni*. Virulence.

[8] Moore J.E., Barton M.D., Blair I.S., Corcoran D., Dooley J.S.G., Fanning S., Kempf I., Lastovica A.J., Lowery C.J., Matsuda M. (2006). The epidemiology of antibiotic resistance in *Campylobacter*. Microbes Infect..

[9] Koenraad P., Rombouts F.M., Notermans S.H.W. (1997). Epidemiological aspects of thermophilic *Campylobacter* in water-related environments: A review. Water Environ. Res..

[10] Frost J.A. (2001). Current epidemiological issues in human campylobacteriosis. J. Appl. Microbiol..

[11] Jokinen C., Edge T.A., Ho S., Koning W., Laing C., Mauro W., Medeiros D., Miller J., Robertson W., Taboada E. (2011). Molecular subtypes of *Campylobacter* spp., *Salmonella enterica*, and *Escherichia coli* O157:H7 isolated from faecal and surface water samples in the Oldman River watershed, Alberta, Canada. Water Res..

[12] Pitkänen T. (2013). Review of *Campylobacter* spp. in drinking and environmental waters. J. Microbiol. Methods.

[13] Rechenburg A., Kistemann T. (2009). Sewage effluent as a source of *Campylobacter* spp in a surface water catchment. Int. J. Environ. Res. Public Health.

[14] Schonberg-Norio D., Takkinen J., Hänninen M.L., Katila M.L., Kaukoranta S.S., Mattila L., Rautelin H. (2004). Swimming and *Campylobacter* infections. Emerg. Inf. Dis..

[15] Arimi S.M., Fricker C.R., Park R.W.A. (1988). Occurrence of thermophilic Campylobacters in sewage and their removal by treatment processes. Epidemiol. Infect..

[16] Jacob J., Stelzer W. (1992). Comparison of 2 media for the isolation of thermophilic Campylobacters from waste-waters of different quality. Zentralbl. Mikrobiol..

[17] Khan I.U.H., Hill S., Nowak E., Edge T.A. (2013). Effect of incubation temperature on the detection of thermophilic *Campylobacter* species from freshwater beaches, nearby wastewater effluents, and bird fecal droppings. Appl. Environ. Microbiol..

[18] Stampi S., Varoli O., Deluca G., Zanetti F. (1992). Occurrence, removal and seasonal-variation of thermophilic Campylobacters in a sewage-treatment plant in Italy. Zentralbl. Hyg. Umweltmed..

[19] Baylis C.L., MacPhee S., Martin K.W., Humphrey T.J., Betts R.P. (2000). Comparison of three enrichment media for the isolation of *Campylobacter* spp. from foods. J. Appl. Microbiol..

[20] Engberg J., On S.L.W., Harrington C.S., Gerner-Smidt P. (2000). Prevalence of *Campylobacter*, *Arcobacter*, *Helicobacter*, and *Sutterella* spp. in human fecal samples as estimated by a reevaluation of isolation methods for Campylobacters. J. Clin. Microbiol..

[21] Musgrove M.T., Berrang M.E., Byrd J.A., Stern N.J., Cox N.A. (2001). Detection of *Campylobacter* spp. in ceca and crops with and without enrichment. Poult. Sci..

[22] Abulreesh H.H., Paget T.A., Goulder R. (2006). *Campylobacter* in waterfowl and aquatic environments: Incidence and methods of detection. Environ. Sci. Technol..

[23] Miller W.G., Mandrell R.E., Ketley J.M., Konkel M.E. (2005). Prevalence of *Campylobacter* in the food and water supply: Incidence, outbreaks, isolation and detection. Campylobacter: Molecular and Cellular Biology.

[24] Jokinen C.C., Koot J.M., Carrillo C.D., Gannon V.P.J., Jardine C.M., Mutschall S.K., Topp E., Taboada E.N. (2012). An enhanced technique combining pre-enrichment and passive filtration increases the isolation efficiency of *Campylobacter jejuni* and *Campylobacter coli* from water and animal fecal samples. J. Microbiol. Methods.

[25] Isolation of Thermotolerant *Campylobacter*—Review & Methods for New Zealand Laboratories. http://www.moh.govt.nz/notebook/nbbooks.nsf/0/73166eb251837f95cc257834000271db/$FILE/IsolationOfThermotolerantCampylobacter.pdf.

[26] (2005). ISO 17995. Water Quality—Detection and Enumeration of Thermotolerant Campylobacter Species.

[27] Ugarte-Ruiz M., Gomez-Barrero S., Porrero M.C., Alvarez J., Garcia M., Comerón M.C., Wassenaar T.M., Dominguez L. (2012). Evaluation of four protocols for the detection and isolation of thermophilic *Campylobacter* from different matrices. J. Appl. Microbiol..

[28] Baliarda A., Faure D., Urdaci M.C. (2002). Development and application of a nested PCR to monitor brood stock salmonid ovarian fluid and spleen for detection of the fish pathogen *Flavobacterium psychrophilum*. J. Appl. Microbiol..

[29] GenBank. http://www.ncbi.nlm.nih.gov/genbank/.

[30] ClustalW2 Multiple Sequence Alignment EMBL-EB. http://www.ebi.ac.uk/Tools/msa/clustalw2/.

[31] EzTaxon-e-EzBioCloud.net. http://www.eztaxon.org/.

[32] EUCAST. http://www.eucast.org/.

[33] Abramson J.H. (2011). WINPEPI updated: Computer programs for epidemiologists, and their teaching potential. Epidemiol. Perspect. Innov..

[34] Cálculo De Prevalencia-Winepi: Working In Epidemiology. http://www.winepi.net/sp/disease/cprev1.asp.

[35] Kiehlbauch J.A., Brenner D.J., Nicholson M.A., Baker C.N., Patton C.M., Steigerwalt A.G., Wachsmuth I.K. (1991). *Campylobacter butzleri sp nov* isolated from humans and animals with diarrheal illness. J. Clin. Microbiol..

[36] Vandamme P., Falsen E., Rossau R., Hoste B., Segers P., Tytgat R., Deley J. (1991). Revision of *Campylobacter*, *Helicobacter*, and *Wolinella* taxonomy-emendation of generic descriptions and proposal of *Arcobacter* gen-nov. Int. J. Syst. Bacteriol..

[37] Josefsen M.H., Lofstrom C., Hansen T.B., Christensen L.S., Olsen J.E., Hoorfar J. (2010). Rapid quantification of viable *Campylobacter* bacteria on chicken carcasses, using Real-Time PCR and propidium monoazide treatment, as a tool for quantitative risk assessment. Appl. Environ. Microbiol..

[38] Jones D.M., Sutcliffe E.M., Curry A. (1991). Recovery of viable but non-culturable *Campylobacter jejuni*. J. Gen. Microbiol..

[39] Rollins D.M., Colwell R.R. (1986). Viable but non culturable stage of *Campylobacter jejuni* and its role in survival in the natural aquatic environment. Appl. Environ. Microbiol..

[40] Rosef O., Kapperud G., Skjerve E. (1987). Comparison of media and filtration procedures for qualitative recovery of thermotolerant *Campylobacter* spp from naturally contaminated surface-water. Int. J. Food Microbiol..

[41] Vereen E., Lowrance R.R., Cole D.J., Lipp E.K. (2007). Distribution and ecology of campylobacters in coastal plain streams (Georgia, United States of America). Appl. Environ. Microbiol..

[42] Nocker A., Sossa-Fernandez P., Burr M.D., Camper A.K. (2007). Use of propidium monoazide for live/dead distinction in microbial ecology. Appl. Environ. Microbiol..

[43] Nogva H.K., Dromtorp S.M., Nissen H., Rudi K. (2003). Ethidium monoazide for DNA-based differentiation of viable and dead bacteria by 5ʹ-nuclease PCR. Biotechniques.

[44] Pacholewicz E., Swart A., Lipman L.J.A., Wagenaar J.A., Havelaar A.H., Duim B. (2013). Propidium monoazide does not fully inhibit the detection of dead *Campylobacter* on broiler chicken carcasses by qPCR. J. Microbiol. Methods.

[45] Seinige D., von Köckritz-Blickwede M., Krischek C., Klein G., Kehrenberg C. (2014). Influencing factors and applicability of the viability EMA-qPCR for a detection and quantification of *Campylobacter* cells from water samples. PLoS ONE.

[46] Newell D.G., Shreeve J.E., Toszeghy M., Domingue G., Bull S., Humphrey T., Mead G. (2001). Changes in the carriage of *Campylobacter* strains by poultry carcasses during processing in abattoirs. Appl. Environ. Microbiol..

[47] Ugarte-Ruiz M., Wassenaar T.M., Gomez-Barrero S., Porrero M.C., Navarro-Gonzalez N., Dominguez L. (2013). The effect of different isolation protocols on detection and molecular characterization of *Campylobacter* from poultry. L. Appl. Microbiol..

[48] Williams L.K., Sait L.C., Cogan T.A., Jorgensen F., Grogono-Thomas R., Humphrey T.J. (2012). Enrichment culture can bias the isolation of *Campylobacter* subtypes. Epidemiol. Infect..

[49] Korhonen L.K., Martikainen P.J. (1991). Comparison of the survival of *Campylobacter jejuni* and *Campylobacter coli* in culturable form in surface-water. Can. J. Microbiol..

[50] Thomas C., Gibson H., Hill D.J., Mabey M. (1999). *Campylobacter* epidemiology: An aquatic perspective. J. Appl. Microbiol..

[51] Meinersmann R.J., Berrang M.E., Little E. (2013). *Campylobacter* spp. recovered from the Upper Oconee river watershed, Georgia in a 4-Year Study. Microb. Ecol..

[52] Khan I.U., Gannon V., Jokinen C.C., Kent R., Koning W., Lapen D.R., Medeiros D., Miller J., Neumann N.F., Phillips R. (2014). A national investigation of the prevalence and diversity of thermophilic *Campylobacter* species in agricultural watersheds in Canada. Water Res..

[53] Jacobs-Reitsma W., Ulrike L., Wagenaar J., Nachamkim I., Szymanski C.M., Blaser M.J. (2008). *Campylobacter* in the food supply. Campylobacter.

[54] Man S.M. (2011). The clinical importance of emerging *Campylobacter* species. Nat. Rev. Gastroenterol. Hepatol..

[55] Baquero F., Martinez J.L., Canton R. (2008). Antibiotics and antibiotic resistance in water environments. Curr. Opin. Biotechnol..

[56] Börjesson S., Matussek A., Melin S., Lofgren S., Lindgren P.E. (2010). Methicillin-resistant *Staphylococcus aureus* (MRSA) in municipal wastewater: An uncharted threat?. J. Appl. Microbiol..

[57] Denis M., Mourand G., Chidaine B., Kempf I. (2012). Susceptibility of *Campylobacter* isolates from river water in Brittany, France. Int. J. Antimicrob. Agents.

[58] Pérez-Boto D., Javier García-Peña F., Carlos Abad-Moreno J., Aurora Echeita M. (2013). Antimicrobial susceptibilities of *Campylobacter jejuni* and *Campylobacter coli* strains isolated from two early stages of poultry production. Microb. Drug Resist..

[59] EFSA (European Food Safety Authority), ECDC (European Centre for Disease Prevention and Control) (2013). The European Union summary report on antimicrobial resistance in zoonotic and indicator bacteria from humans, animals and food in 2011. EFSA J..

[60] González A., Botella S., Montes R.M., Moreno Y., Ferrus M.A. (2007). Direct detection and identification of *Arcobacter* species by multiplex PCR in chicken and wastewater samples from Spain. J. Food Protect..

[61] Son I., Englen M.D., Berrang M.E., Fedorka-Cray P.J., Harrison M.A. (2007). Prevalence of *Arcobacter* and *Campylobacter* on broiler carcasses during processing. Int. J. Food Microbiol..

[62] Moreno Y., Botella S., Alonso J.L., Ferrus M.A., Hernandez M., Hernandez J. (2003). Specific detection of *Arcobacter* and *Campylobacter* strains in water and sewage by PCR and fluorescent *in situ* hybridization. Appl. Environ. Microbiol..

[63] Lee C., Agidi S., Marion J.W., Lee J. (2012). *Arcobacter* in Lake Erie Beach Waters: An emerging gastrointestinal pathogen linked with human-associated fecal contamination. Appl. Environ. Microbiol..

[64] Rahimi E. (2014). Prevalence and antimicrobial resistance of *Arcobacter* species isolated from poultry meat in Iran. Br. Poult. Sci..

